# Off-Licence Antipsychotic Prescribing and Physical Health Monitoring in Inpatients With a Diagnosis of Personality Disorder (Borderline Type)

**DOI:** 10.1192/bjo.2026.11709

**Published:** 2026-06-30

**Authors:** Lazaros Hadjiforados, James Fallon, Poppy J. Riddle

**Affiliations:** 1 Brighton and Sussex Medical School, Brighton, United Kingdom; 2 Sussex Partnership NHS Foundation Trust, Brighton, United Kingdom

## Abstract

**Aims:**

Personality disorder (borderline type), previously known as Emotionally Unstable Personality Disorder (EUPD), is a complex psychiatric condition associated with unstable relationships, emotional lability and transient psychotic-like features. National Institute for Health and Care Excellence (NICE) guidelines for EUPD (CG78) advise against routine use of psychiatric medication for personality disorder symptoms, recommending pharmacological treatment only for comorbid mental illness in accordance with relevant guidance. Antipsychotics are not licensed for EUPD. Where used, prescribing should be short-term, crisis-focused, and accompanied by clear documentation and informed consent. However, national data from the Prescribing Observatory for Mental Health indicate that antipsychotic prescribing in EUPD often occurs off-licence and may continue long-term by default, with variable documentation and review. This audit evaluated antipsychotic prescribing practices for inpatients with EUPD within Sussex Partnership NHS Foundation Trust, focusing on documentation of prescribing rationale, communication with patients regarding above license and physical health monitoring.

**Methods:**

A retrospective review of clinical records was conducted across all working-age adult inpatient wards within the Trust. Data were collected as cross-sectional snapshots at three time points, each one month apart. Inpatients aged 18-65 years with a documented diagnosis of EUPD were included. Patients with primary psychotic disorder and out-of-area admissions were excluded. Where diagnostic ambiguity existed, a senior psychiatrist reviewed clinical notes to determine eligibility on a case-by-case basis. Outcomes were assessed against NICE and local Trust guidelines using descriptive statistics.

**Results:**

Of 508 records reviewed, 52 patients met the inclusion criteria. Antipsychotics were prescribed in 86.5% (45/52) of patients, with 20% (9/45) initiated during admission and 8.9% (4/45) receiving polypharmacy. None of the antipsychotics initiated were documented as time-limited. Documentation of communication regarding off-licence prescribing was present in only 6.6% (3/45) of cases. Physical health monitoring was inconsistently completed: body mass index was tracked in 80.7% (42/52) patients, HbA1c in 21.1% (11/52), electrocardiogram in 48.1% (25/52), blood pressure in 98.1% (51/52), prolactin in 42.3% (25/52), and cholesterol in 53.8% (28/52). Random glucose was requested instead of HbA1c in 42.3% (22/52) of cases, contrary to Trust guidelines.

**Conclusion:**

Antipsychotic prescribing for EUPD inpatients frequently deviated from guideline recommendations, with significant gaps in documentation, consent recording, and physical health monitoring. These findings highlight the need for improved adherence to guidance, clearer documentation standards, and structured monitoring pathways to enhance patient safety and quality of care.

